# The transition from acute to chronic pain: might intensive care unit patients be at risk?

**DOI:** 10.1186/2110-5820-2-36

**Published:** 2012-08-16

**Authors:** Maria Kyranou, Kathleen Puntillo

**Affiliations:** 1 Office of Nursing Education and Research, Papageorgiou Hospital, West Ring Road, Nea Efkarpia, 56429 Thessaloniki, Greece; 2 Department of Physiological Nursing, University of California San Francisco, 2 Koret Way, San Francisco, CA 94143

**Keywords:** Pain, Acute, Chronic, Acute-to-chronic, Intensive care unit, Critical care, Nerve sensitization

## Abstract

Pain remains a significant problem for patients hospitalized in intensive care units (ICUs). As research has shown, for some of these patients pain might even persist after discharge and become chronic. Exposure to intense pain and stress during medical and nursing procedures could be a risk factor that contributes to the transition from acute to chronic pain, which is a major disruption of the pain neurological system. New evidence suggests that physiological alterations contributing to chronic pain states take place both in the peripheral and central nervous systems. The purpose of this paper is to: 1) review cutting-edge theories regarding pain and mechanisms that underlie the transition from acute to chronic pain, such as increases in membrane excitability of peripheral and central nerve fibers, synaptic plasticity, and loss of the function of descending inhibitory pain fibers; 2) provide information on the association between the immune system and pain and its crucial contribution to development of chronic pain syndromes, and 3) discuss mechanisms at brain levels in the nervous system and their contribution to affective (i.e., emotional) states associated with chronic pain conditions. Finally, we will offer suggestions for ICU clinical interventions to attempt to prevent the transition from acute to chronic pain.

## Review

Most, if not all, patients in intensive care units (ICUs) will experience pain at some point during their ICU stay related to their injury, surgery, burns, or comorbidities, such as cancer,and/or from the myriad procedures performed for diagnostic or treatment purposes [1-4]. Indeed, even medical patients experience substantial pain at rest [5]. Despite increased attention to assessment and pain management,pain remains a significant problem for ICU patients [1,6-8].

Unrelieved pain in adult ICU patients is far from benign. Medical and surgical ICU patients who recalled pain and other traumatic situations while in the ICU (27% of 80 patients) had a higher incidence of chronic pain and posttraumatic stress disorder symptoms than did a comparative group of ICU patients [9]. Indeed, concurrent or past pain may be the greatest risk factor for development of chronic pain [4,10]. Despite clinical concerns about the contribution of ICU hospitalization to development of chronic pain, a systematic review of the literature on quality of life (QOL) after hospitalization in an ICU concluded that chronic pain in ICU survivors did not differ from a matched normal population [11], except in patients with acute respiratory distress syndrome(ARDS) [12].

Nevertheless, recent findings from studies in various ICU patient populations as well as with longer follow-up periods are in keeping with the findings for chronic pain in patients with ARDS. Patients discharged from a medical-surgical ICU reported higher bodily pain at 12 months after hospitalization compared with 3 months [13]. For patients with burns hospitalized in an ICU, the majority of responders (79% of patients) suffered from moderate to severe pain 1 year after the injury [14]. Furthermore, a group of German researchers found highly significant differences in the pain intensity and pain interference between survivors of severe sepsis compared with a healthy German population [15]. However, others have not confirmed these findings in ICU survivors of sepsis in the Netherlands [16].

When the follow-up of the patients after hospitalization in an ICU was even longer, such as 2 years after major surgery, almost 60% of patients reported having moderate or extreme problems in usual activities: 56% had pain, and 56% had mobility problems [17]. Similarly, patients hospitalized in a medical-surgical ICU had higher bodily pain reports 5 years after discharge compared with 3 months [18]. Importantly, according to the findings of a recent study with a mean follow-up of 8 (range, 6–11) years, nearly half of all patients hospitalized in an ICU for all surgical classifications combined still had problems with cognition (43%), mobility (52%), and pain/discomfort (57%) [19].

Despite methodological problems with these studies, mainly small sample of patients, no homogeneity of pain measures, and high mortality rate or loss to follow-up, the observations for severe functional limitations combined with high reports of pain even years after ICU discharge grant further exploration. Important questions to ask are: Why does acute pain transition into a chronic pain state in some patients? Might medical and/or nursing procedures performed during the ICU stay, or other painful stimuli experienced by patients in ICUs, be contributing factors to the neurological mechanism of the transition?

Today, we know more about the physiological mechanisms of acute pain as well as mechanisms of acute-to-chronic pain. Therefore, the purpose of this paper is to emphasize the risk that exists for ICU patients and others in developing chronic pain as a result of an acute pain experience. To do so, we will: 1) review cutting-edge theories regarding pain and mechanisms that underlie the transition from acute to chronic pain, such as increases in membrane excitability of peripheral and central nerve fibers, synaptic plasticity, and loss of the function of descending inhibitory pain fibers; 2) provide information on the association between the immune system and pain and its crucial contribution to development of chronic pain syndromes; and 3) discuss mechanisms at supraspinal levels in the nervous system and their contribution to affective (i.e., emotional) states associated with chronic pain conditions. As we address these three issues, we will be able to offer suggestions for clinical interventions to attempt to prevent the transition from acute to chronic pain.

### Terminology used to describe the phases of the transition from acute to chronic pain

The International Association for the Study of Pain (IASP) describes pain as “an unpleasant sensory and emotional experience associated with actual or potential tissue damage, or described in terms of such damage” [20]. Acute pain can arise from cutaneous (i.e., from the skin), deep somatic (i.e., from muscle, bone) or visceral structures (i.e., from organs within the chest and abdomen) [21]. When unexpected exposure to potentially harmful stimuli occurs, pain manifests as an automatic (reflex) withdrawal response along with a motivational reaction, most frequently a feeling of unpleasantness (i.e., a negative affect) [22]. The sensory process of detecting the “actual or potential tissue damage” is called **nociception** and clinically manifests as hypersensitivity to mechanical (e.g., pressure applied to an abdominal incision during coughing), thermal (e.g., heat from burns), or chemical stimuli (e.g., when inflammatory substances are released during an injury). (See Table 1 for definitions of **hypersensitivity** and other terms used in this review.) The hypersensitivity at the site of the injury that is associated with **acute pain** motivates the patient to avoid further damage [23,24] and, thus, is protective of tissues and the organism [25]. Yet, this hypersensitivity may cause delayed mobilization if the ICU patient is in too much pain [26]. In the context of injury (e.g., trauma, surgery), acute pain might persist for a few hours or days or even for up to several months and is still considered acute [27].

**Table 1:** Explanation of pain terminology

	
**Acute pain**	Intensely discomforting, distressful, or agonizing sensation associated with trauma or disease, with well-defined location, character, and timing [20]). Year introduced: 2012.
**Allodynia**	Pain due to a stimulus that does not normally provoke pain [20].
**Central sensitization**	Increased responsiveness of nociceptive neurons in the central nervous system to their normal or subthreshold afferent input [20].
**Chronic pain**	Pain that continues or recurs over a prolonged period, caused by various diseases or abnormal conditions [28].
**Hyperalgesia**	Increased pain from a stimulus that normally provokes pain [20].
**Hypersensitivity or hyperesthesia**	Increased sensitivity to stimulation, excluding the senses [20].
**Long-term potentiation (LTP)**	A long-lasting strengthening of the response of a postsynaptic nerve cell to stimulation across the synapse that occurs with repeated stimulation and is thought to be related to learning and long-term memory [29].
**Modulation**	The inhibition or facilitation of pain [30].
**Neuropathic pain**	Pain caused by a lesion or disease of the somatosensory nervous system [20].
**Pain affect**	Feeling or emotion related to pain, especially as manifested by facial expression or body language [31].
**Pain sensation**	An unpleasant sensory experience associated with actual or potential tissue damage, or described in terms of such damage [20].
**Peripheral sensitization**	Increased responsiveness and reduced threshold of nociceptive neurons in the periphery to the stimulation of their receptive fields [32].
**Sensitization**	An increased response to stimulation that is mediated by amplification of signaling [32].
**Supraspinal**	Situated above the vertebral column [31].
**Synaptic plasticity**	The ability of the connection, or synapse, between two neurons to change in strength in response to use or disuse of transmission over synaptic pathways [33].

**Peripheral sensitization** is usually the result of exposure of nociceptors (i.e., receptors that respond to a noxious stimulus) to inflammatory products and mediators of tissue injury. This subsequently contributes to reductions in nerve thresholds at nociceptive terminals, increases in sensitivity, and amplification of the excitability of the peripheral afferent nerve fibers [32]. Whereas this action can last for an extended period of time, it is not permanent [34]. Under normal circumstances, peripheral hypersensitivity returns to normal when inflammation subsides or the source of the injury is removed. Consider, for example, the sensitivity of a surgical incision that decreases over a matter of days.

However, in certain instances, pain can exceed the average period of healing, cease serving any apparent protective function, and become **chronic** (i.e., after 2 to 3 months) [34,35]. In these cases, peripheral hypersensitivity does not return to normal. On the contrary, it indirectly contributes to the initiation of central sensitization; i.e., sensitization of the spinal cord nerves. During **central sensitization**, nociceptive-specific neurons may progressively increase their response to repeated nonpainful stimuli, develop spontaneous activity, and increase the area of the body that is involved with the pain [36]. The **hyperalgesia** of central sensitization usually develops as part of ongoing pathology (i.e., damage to peripheral or central nerve fibers, cancer, rheumatoid arthritis) and is considered maladaptive. Furthermore, hyperalgesia can be induced by opioid administration and/or interruption, although this phenomenon is not well understood yet [37]. Judicious treatment of pain in the ICU may help to preempt development of central sensitization. This is important, because once this type of sensitization occurs for prolonged periods, it can be maintained by lower or different kind of inputs to the central nervous system (CNS). This is identified as **neuropathic pain**, or pain caused by damage to peripheral or central nerve fibers themselves.

### Mechanisms involved in the transition from acute to chronic pain increases in membrane excitability of peripheral nerve fibers

Peripheral neurons that respond to noxious stimuli and serve to detect potentially harmful thermal, mechanical, or chemical stimuli are called nociceptors [38]. There are two main types of nociceptors: medium-diameter myelinated Aδ afferents that mediate acute pain that occurs quickly and is well localized; and small-diameter unmyelinated C fibers that initiate a slightly delayed and more diffuse pain [39].

Increases in the membrane excitability of peripheral nociceptors most commonly result from inflammation-associated changes in the chemical environment of the nerve fiber [40]. Activated nociceptors as well as nonneuronal cells that reside within, or infiltrate into, the injured area (e.g., mast cells, macrophages, platelets, endothelial cells) release a variety of molecules, including neuropeptides [e.g., substance P, calcitonin gene-related peptide (CGRP), bradykinin], neutrophins, cytokines, and eicosanoids or related lipids (e.g., prostaglandins, thromboxanes, and leukotrienes). These substances bind to receptors on peripheral nociceptive terminals, which leads to heightened excitability of the nerve fiber.

In addition, the release of numerous cytokines, including interleukin-1β (IL-1β), IL-6, and tumor necrosis factor α (TNF-α) [41], activates the immune system which, in turn, may affect neuronal function and increase pain responses. As evidence of this, administration of a proinflammatory cytokine antagonist immediately after peripheral nerve injury or inflammation reduces pain responses [42-45]. The number of macrophages that are present at a site of injury has directly correlated with the severity of neuropathic pain [46,47]. ICU clinicians can attempt to decrease the effects of these inflammatory mediators of pain by considering the use of anti-inflammatory agents, such as indomethacin, as an adjunctive therapy to other pain medications if the patient has no contraindications to their use [48]. Contradictions can include renal insufficiency, active peptic ulcer disease, and coagulation problems [49].

### Increases in membrane excitability of dorsal root ganglia

Nociceptors have peripheral axonal branches that convey information from the periphery to ganglia outside the spinal cord and central axonal branches that convey information from the ganglia to the spinal cord. The cell bodies of peripheral nociceptors are located in dorsal root ganglia (DRG) for stimuli originating in the body and in the trigeminal ganglion for stimuli from the face and mouth. Following nerve injury and in the setting of inflammation, primary sensory neurons become hyperexcitable, altering the organization of DRG neurons [50]. In addition, the DRG contains a variety of immune and immune-like cells (e.g., endothelial cells, dendritic cells, glially derived) that exist in close proximity to each DRG neuronal cell body [51]. In response to peripheral nerve damage and/or inflammation, the above nonneuronal cells, as well as immune cells, drawn into the DRG from the circulation, release proinflammatory cytokines and growth factors [52]. These responses contribute to the upregulation of cytokine receptors in DRG neurons [53-55], which leads to the release of substance P [56] and calcitonin gene-related peptide [57], which are pain-generating substances. Gradually, these events lead to DRG membrane depolarizations, and nociceptors start firing nociceptive signals from the DRG to the spinal cord at increased frequency [34]. Preventing this inflammatory cascade may keep these normally quiet cells from firing [58].

### Synaptic plasticity in the spinal cord

**Figure 1:**
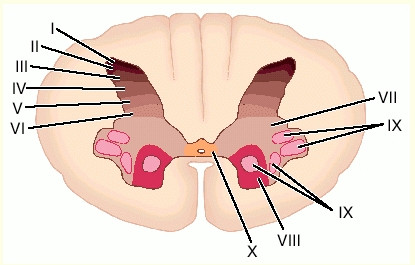
Rexed's laminae in a cross-section of the spinal cord at approximately the level of the seventh cervical vertebra (C7) with permission.

The outer area within spinal cord consists of white matter, and the inner area is composed of gray matter. These areas comprise a busy milieu of nerve fiber transmission. The white matter contains ascending and descending neuronal tracts. The grey matter contains ten different layers, known as the Rexed laminae, on the basis of the characteristics of their neurons. The dorsal horn (posterior), where primary nociceptive afferent nerve fibers project, contains laminae I to VI, whereas the ventral horn (anterior), comprising the motor neurons, contains laminae VII to IX. Lamina X surrounds the central canal (Figure 1).

Neurons in lamina I receive direct synaptic input from Aδ and C nociceptive fibers and respond exclusively to nociceptive stimulation. Lamina II, also called the substantia gelatinosa, is made up almost exclusively of interneurons (both excitatory and inhibitory). Some of these interneurons respond only to nociceptive inputs, whereas others respond to both nociceptive and non-nociceptive stimuli [38]. Lamina III and IV contain neurons that receive input from Aβ fibers that respond predominantly to nonnoxious stimuli (e.g., touch). Importantly, lamina V contains primarily neurons that respond to a wide range of stimulus intensities as well as a combination of inputs (i.e., nociceptive and nonnociceptive, from skin, muscle, and viscera). These neurons are called wide-dynamic-range (WDR) neurons. WDR neurons receive input from Aβ and Aδ fibers as well as from C fibers, either directly on their dendrites or indirectly via excitatory interneurons that themselves receive input from peripheral C fibers [38]. Neurons in lamina VI receive input from nociceptors that mediate “fast pain” activating ascending pathways that will lead to the perception of pain as well as motor neurons involved in the withdrawal reflex (the automatic withdrawal of an extremity from a painful stimulus) [59].

During exposure to injury and subsequent activation of peripheral stimuli, the central terminals of these spinal cord nociceptors release a number of neurotransmitters (e.g., glutamate, substance P, brain-derived neurotrophic factor (BDNF), and calcitonin gene-related peptide (CGRP)), which bind to receptors of postsynaptic neurons in the dorsal horn spinal cord’s lamina I. When the exposure is brief and not caused by any peripheral damage, the *N*-methyl *D*-aspartate (NMDA) subtype of the glutamate receptor is not activated. However, when a repetitive and/or high-frequency stimulation of C-fibers occurs, it is likely that the release of glutamate and other substances leads to a prolonged, slow depolarization of the neuron, and subsequent removal of the NMDA block. When the NMDA receptor becomes unblocked, a large influx of calcium ions (Ca^2+^) is allowed into the postsynaptic neuron. Once inside the cell, Ca^2+^ promotes transcriptional changes that subsequently contribute to the maintenance of nerve sensitization [34].

In an attempt to prevent development of this sensitization, which prolongs pain, ICU clinicians can consider use of dual pharmacological therapies in certain situations. For instance, mechanically ventilated patients with Guillain-Barré syndrome and neuropathic pain can receive superior pain relief from opioids plus oral gabapentin or carbamazepine, which are both categorized as antiepileptic and antihyperalgesic drugs compared with the use of opioids alone [60,61]. In fact, gabapentin may exert a selective effect on the nociceptive process involved in central neuronal sensitization, which is the rationale for its use in the treatment of acute postoperative pain [62]. In addition, ketamine is an NMDA-receptor antagonist that has been demonstrated to prevent hyperalgesia in postoperative major abdominal surgery patients [63]. The effectiveness of ketamine for ICU patients requires further study.

Changes that occur to nerve synapses in dorsal horn neurons as a result of stimulation that goes unblocked are frequently compared with **long-term potentiation (LTP)**. LTP is a process that occurs in the cortex and leads to the formation of long-term memory through changes in **synaptic plasticity**[38]. The major synaptic alteration that contributes to central sensitization and ongoing pain is when increased activity in one set of synapses results in the facilitation of the activity in another set of synapses [34]. Long-term potentiation is responsible for the major sensory manifestations associated with central sensitization. One manifestation is **allodynia**, which is when a person experiences pain in a localized area that is not due to a painful stimulus but, rather, a nonpainful stimulus, such as touch. A second manifestation is called **secondary hyperalgesia**, which is an increase in pain sensitivity in noninjured areas beyond the area of primary injury [36]. It is likely that the phenomenon of central sensitization observed in pathologic clinical states includes both of the above processes [34,64,65]. If this is the case, avoiding LTP through analgesic control of acute pain is an important goal.

### Changes in inhibitory control at the level of the spinal cord

When a nociceptive signal from the periphery reaches the spinal cord, it is relayed to projection neurons, which carry the signal along ascending pathways in the spinal cord white matter to higher centers in the brain. At the same time, inhibitory interneurons interact with the terminals of primary nociceptors or projection neurons to maintain the orderly processing of sensory information [30,66]. The inhibitory function of interneurons in the spinal cord is mediated by the release of inhibitory neurotransmitters, such as γ-aminobutyric acid (GABA) and glycine. Persistent nociceptive stimulation caused by inflammation and/or neuropathy can affect neurotransmission at this level in three ways: 1) by decreasing the number of sites that release GABA and glycine; 2) by decreasing the number of receptors to which they bind; and 3) by increasing the speed by which the inhibitory neurotransmitters are removed from the synaptic cleft [67,68]. Thus, the disrupted function of interneurons due to loss of inhibitory control and increased stimulation could contribute to the cascade of events that lead to persistent chronic pain. Opioids, with mu-receptors in the spinal cord, can help to maintain an inhibitory effect. Indeed, opioids are the most frequently used analgesics in ICUs, and IV opioids, such as fentanyl, hydromorphone, and remifentanil, can be considered the first-line drug class of choice to treat nonneuropathic pain in critically ill patients [69]. However, follow-up of these patients is required for the early recognition of “opioid-induced hyperalgesia,” a paradoxical hyperalgesic state induced after the administration or abrupt cessation of high opioid doses. Studies of patients with chronic pain receiving opioid therapy as well as chronic users of methadone support enhanced pain perception during laboratory pain tests [37]. Nevertheless, due to the experimental setting of these studies and the lack of evidence in the ICU setting, further research is necessary to determine the presence of opioid-induced hyperalgesia in ICU patients.

### Changes in descending modulation

**Figure 2:**
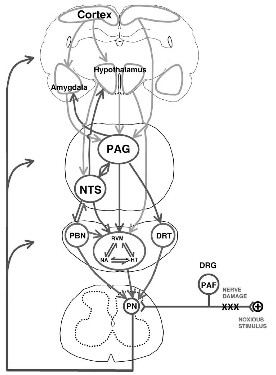
** Cerebral structures involved in the descending modulation of nociceptive information.** Amyg, amygdala; CX, cortex; DRG, dorsal root ganglion; DRT, dorsoreticular nucleus; Hypothal, hypothalamus; NA, noradrenaline; NTS, nucleus tractus solitarius; PAF, primary afferent fibre; PBN, parabrachial nucleus; PAG, periaqueductal grey; Perikarya 5-HT, serotonergic perikarya; PN, projection neurones; RVM, rostroventral medulla [30] (permission granted).

Under normal circumstances, dorsal horn spinal cord neurons as well as the central terminals of primary afferent fibers receive additional inhibitory input from fibers that descend from supraspinal structures such as the cortex, midbrain, and brain stem to the spinal cord [30,70-72]. For example, fibers that descend from a midbrain structure called the periaqueductal grey (PAG) (Figure 2) activate serotoninergic neurons in the rostroventral medulla (RVM) or noradrenergic neurons in the reticular formation in the pons. When these descending inhibitory neurons reach the spinal cord, they release serotonin and noradrenaline. These substances act directly or indirectly through other inhibitory interneurons to inhibit the release of noxious transmitters from primary afferent fibers or to inhibit the activation of spinal cord neurons that project a noxious stimulus to the brain [30]. Tramadol has been recommended for consideration for mild pain, because it is both a mu-receptor agonist and a serotonin and noradrenaline reuptake inhibitor [69]. Inhibition of reuptake inhibitors serotonin and noradrenaline maintains their prevention of nociceptive transmission. Likewise, nefopam is a nonopioid analgesic that inhibits dopamine, norepinephrine, and serotonin reuptake and, thus, prevents nociceptive transmission. Its effectiveness on moderate pain in a sample of 59 ICU patients has been demonstrated, although some patients experienced an increase in heart rate and decrease in mean arterial pressure [73].

Of note, the RVM in the medulla also is an important relay station for descending facilitatory influences on nociceptive spinal transmission [74,75]. More specifically, sustained ascending nociceptive input can activate descending pain f*acilitatory* systems from the rostroventromedial medulla (RVM) through the release of pronociceptive excitatory neurotransmitters. Thus, persistence of pain for long periods could potentially lead to more pain due to top-down effects on the spinal cord, another reason for treating pain as aggressively as possible in ICU patients.

### Immune to nervous system interactions in the CNS and pain facilitation

The “sickness response” is described as the constellation of fever, increased sleep, decreased activity, and pain facilitation (i.e., sickness-induced hyperalgesia) [76]. Spinal cord immune (i.e., glia) cells have been shown to participate in pain enhancement as part of the adaptive sickness response; thus, they could be involved in pathological pain states. Studies using animal models of inflammation, peripheral nerve injury, bone cancer pain, and spinal cord injury have shown that trauma or injury to peripheral nerves leads to activation of these immune cells in the CNS [77-84].

Glia cells are numerous within the CNS, and recent evidence suggests that neurons and glia cells constitute a very important unit in the CNS [85]. Proinflammatory cytokines released in the periphery transmit signals through the *blood–brain barrier* to central structures where they can activate nociceptive neurons [86]. Furthermore, immune activation in the periphery can be transferred by way of the vagus and glossopharyngeal nerves [87,88]. These two nerves relay information directly to the nucleus of the solitary tract, or NTS (nucleus tractus solitarii) and ventromedial medulla rather than passing through the spinal cord. These central structures can activate nociceptive neurons of the brainstem and give rise to the final branch of the sickness-induced hyperalgesia pathway, which consists of descending facilitatory fibers that target glia cells in the spinal cord. Activation of glia cells leads to the release of proinflammatory cytokines within the CNS, which bind to membrane receptors expressed by pain-responsive dorsal horn neurons, increasing their excitability [89-91]. Finally, one set of activated glial cells can activate another set of glial cells, which augments nociceptive activation. In short, there is a positive feedback mechanism that intensifies and perpetuates pain. Glial cells become activated during both acute inflammation [92,93] and peripheral nerve damage, which can lead to the development of neuropathic pain [94].

### Central sensitization in brain areas

**Figure 3:**
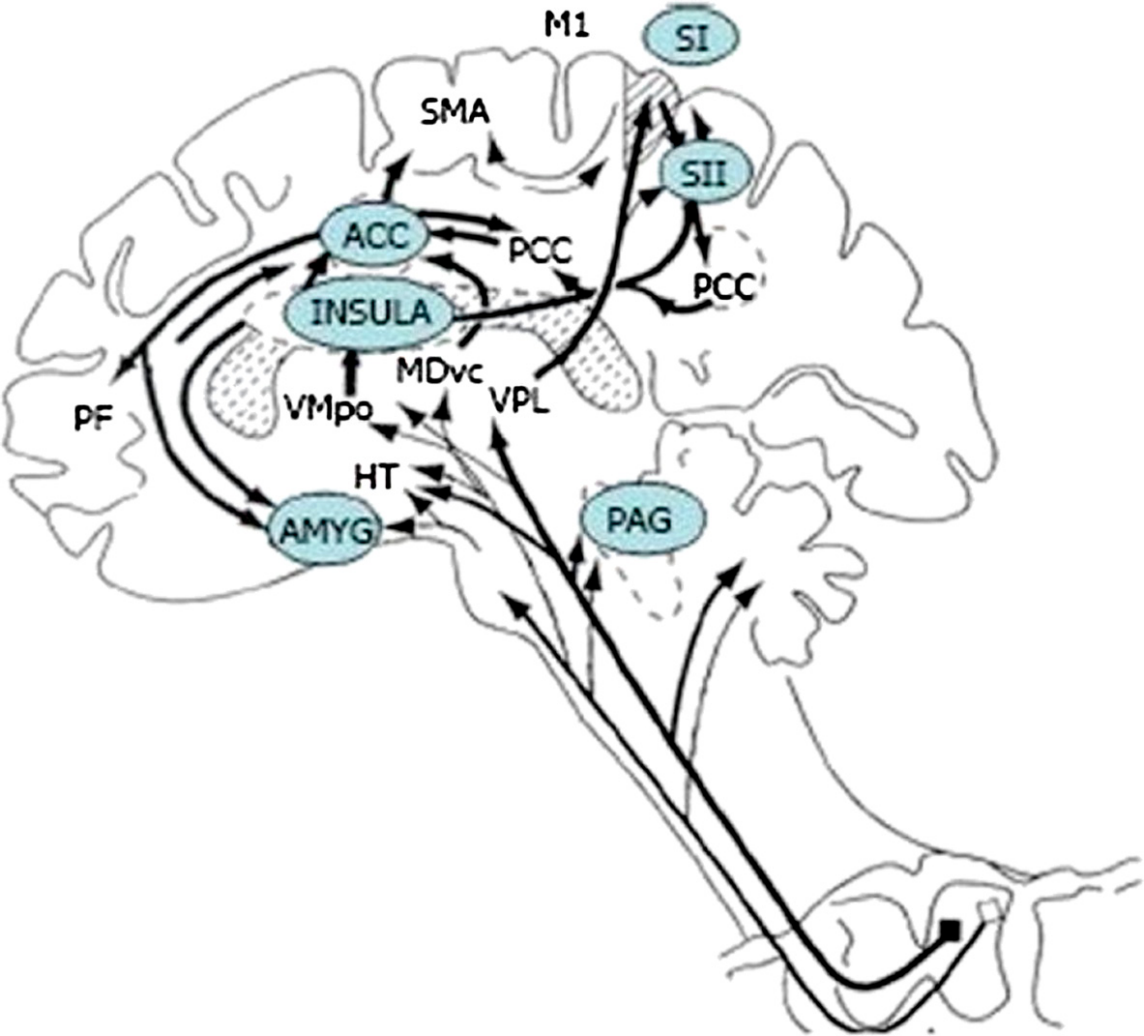
** Supraspinal areas involved in the modulation of pain.** ACC, anterior cingulate cortex; AMYG, amygdala; HT, hypothalamus; M1, motor cortex; MDvc, ventrocaudal part of the medial thalamic dorsal nucleus; PAG, periaqueductal grey; PB, parabrachial nucleus of the dorsolateral pons; PCC, posterior cingulate cortex; PPC, posterior parietal complex; PF, prefrontal cortex; S1, S2, first and second somatosensory cortical areas, respectively; SMA, supplementary motor area; VMpo, ventromedial part of the posterior thalamic nuclear complex; VPL, ventroposterior lateral thalamic nucleus[95] (permission granted).

The perception of pain during both acute injury and in chronic pain states undergoes substantial processing at supraspinal levels (Figure 3) and involves many brain areas. A number of nociceptive pathways project from the spinal cord dorsal horn directly to brainstem and limbic system areas. These pathways directly activate brain structures involved in rudimentary emotional responses to pain, such as autonomic nervous system (ANS) activation, escape, motor responses, arousal, and fear, which require a minimum amount of cognition [95].

A major pathway through which nociceptive input reaches the brain is the lateral spinothalamic tract. This tract projects from the spinal cord to the thalamus and from there to limbic cortical areas, such as the amygdala, the anterior cingulate cortex (ACC), and insular cortex (IC or insula). Another component of the spinothalamic tract projects to somatosensory nuclei of the thalamus, which relays nociceptive information to somatosensory (S-1 and S-2) cortices [96]. This pathway is implicated in the appreciation of the intensity and quality of pain sensations [95]. S1 cortex is generally associated with sensory-discriminative aspects of pain, such as pain intensity and location [97]; S2 cortex likely has additional affective/cognitive functions.

There is a large matrix of pathways and brain areas that work together to influence the pain experience. Some neuronal pathways from S-1/S-2 cortices extend to posterior parietal cortical areas (PPC) and IC, and from IC to ACC, the amygdala, and hippocampus [98]. The IC and ACC are important for affective-motivational and certain cognitive aspects of pain, including attention, anticipation, and evaluation [99-101]. Posterior parietal cortical areas (PPC) integrate somatosensory input with other sensory modalities, such as learning and memory [98,102]. Thus, pain is a whole brain experience.

The convergence of pain pathways that occurs in the brain provides support for a mechanism whereby multiple neural sources, including the limbic system, contribute to pain *affect*; i.e., the emotional component of pain. Convergence of neural pathways at the level of the limbic system would be consistent with a mechanism in which somatic perceptual and cognitive features of pain would be integrated with attentional and emotion mechanisms. Thus, the limbic system may have a complex pivotal role in interrelating (facilitating or inhibiting) attentional and evaluative functions with that of establishing response priorities [102,103].

Sensory and affective dimensions of pain were, until recently, believed to be the result of neural processing in separate but parallel neuronal pathways and brain centers. A more current view proposes that the sensory and affective experience of pain is the result of processing that occurs in both serial *and* parallel ways [95]. That is, regions of the brain involved in somatosensory processing also are important for the processing of the affective dimension of pain. This knowledge could be particularly useful to explore the transition from acute to chronic pain,because the neurobiological dysregulation, manifested at various levels of human functioning (e.g., behavioral, affective, sensory), could open a window on the sequence of events that leads to the establishment of chronic pain. If pain persists over a long period of time, response priorities might change. Pain unpleasantness endured over time engages prefrontal cortical areas involved in reflection and rumination over the future implications of a persistent pain condition [95]. These reflections usually involve perceived interference with one’s body, fear for loss of regular activity, and possible difficulties with enduring pain over time; i.e., after discharge from an ICU [17-19]. In fact, patients with chronic pain are known to have higher postoperative pain scores and a longer time to pain resolution after surgery. Thus, identifying and treating their pain aggressively after surgery could positively impact their long-term pain state [104].

### The prefrontal cortex (PFC)

The prefrontal cortex plays an important role in higher order cognitive functions, such as planning, decision-making, reward expectancy, avoidance of risky choices, and goal-directed behaviors, in both animals and humans [105]. It receives major input from limbic structures, such as the amygdala, and is considered an important area for value-based decision-making [106,107]. Although limbic structures, such as amygdala, ACC, and insula, appear to be important to effect cognitive and emotional factors of pain, they themselves seem to be governed by the prefrontal cortex [108]. Consistent with these observations, the brain area most frequently activated in chronic pain patients is the prefrontal cortex [109]. It is currently believed that, in normal situations, the prefrontal cortex and a network of other interconnected neural structures exert an inhibitory influence on subcortical activation associated with ANS activation and escape behaviors [110]. For example, when a threatening stimulus, such as perceived pain, alarms the subcortical network, the prefrontal cortex diminishes the inhibitory control to allow for sympathetic system activation and escape behaviors. These responses and behaviors may help to account for increases in heart rate and blood pressure and facial expressions observed by ICU clinicians during a patient’s pain experience. However, vital signs cannot be used alone to determine whether pain is present, because they are insensitive measures of pain [111,112].

In the case of excessive activation of this inhibitory network in the prefrontal cortex and in other interconnected neural structures activated in the context of persistent pain, the system might become dysregulated and even disrupted. Such a neurophysiological state could manifest itself as attentional, affective, and/or autonomic dysregulation [110]. In fact, sometimes patients with chronic pain are challenged by affective disorders (i.e., depression, anxiety), exhibit avoidance fears and responses, and develop behaviors that do not have any obvious adaptive character [113]. Thus, effectively managing acute pain in ICU patients may help to prevent the development of affective disorders that often accompany chronic pain.

## Conclusions

Long-term potentiation of synaptic responses in combination with disinhibition/facilitation of descending modulatory endogenous pain circuits and the immune system’s activation seem to be critical in the development and maintenance of conditions of central sensitization and chronic pain. Initially, these alterations were observed at the level of the spinal cord. Interestingly, similar changes are currently being observed as involving several brain areas associated with sensory perception, transmission, modulation, and memory of pain [114]. At the same time, altered emotional and cognitive processing is considered to be an important contributor to the excessive suffering that is a hallmark of chronic pain conditions [115]. Because the pain experience comprises both sensory and emotional components, as presented earlier in the definition of pain by IASP [20], some theorize that physiological alterations that are allowed to persist may be associated with augmentation in the perceptual, affective, and/or motivational components of the pain experience [95,109,113].

Although evidence about transition from acute to chronic pain is new and still evolving and observations are mainly derived from experiments under controlled laboratory settings, this evidence is creating new areas for exploration. Of particular concern is the need to better understand physiological modifications that may occur during the transition of a patient from acute to chronic pain and to prevent this transition as much as possible. Ultimately, the goal in critical care is to appreciate the potential for this transition and attempt to preempt it by aggressive use of therapeutic interventions for pain. In addition, when an ICU patient is recognized as having chronic pain along with their acute condition, clinicians can appreciate the complexity of this condition and use interventions that move toward targeting neuropathic pain mechanisms and/or prevention of opioid-induced hyperalgesia [10,37]. Future basic and clinical research is warranted that tests interventions that could be used to modulate both the somatic and the affective dimension of the acute pain experience and potentially prevent the transition from acute-to-chronic pain.

## Abbreviations

ACC: Anterior cingulate cortex; Amyg: Amygdala; ANS: Autonomic nervous system; ARDS: Acute respiratory distress syndrome; BDNF: Brain-derived neurotrophic factor; CGRP: Calcitonin gene-related peptide; CNS: Central nervous system; CX: Cortex; DRG: Dorsal root ganglia; DRT: Dorsoreticular nucleus; GABA: γ-Aminobutyric acid; Hypothal: Hypothalamus; IASP: International association for the study of pain; IC: Insular cortex or insula; ICU: Intensive care unit; IL-1β: Interleukin-1β; IL-6: Interleukin-6; LTP: Long-term potentiation; mGluR I: Glutamate Receptor subtype I; NA: Noradrenaline; NMDA: N-methyl D-aspartate sub-type of the glutamate receptor; NTS: Nucleus tractus solitarius; PAF: Primary afferent fibre; PAG: Periaqueductal grey; PBN: Parabrachial nucleus; PFC: Prefrontal cortex; PPC: Posterior parietal cortex; RVM: Rostroventral medulla; S-1: S-2: Somatosensory cortices; TNF-α: Tumor necrosis factor α; WDR neurons: Wide dynamic range neurons.

## Competing interests

The authors declare that they have no competing interests.

## Authors' contributions

MK developed the initial draft of this manuscript as part of her PhD dissertation. KP was MK’s dissertation advisor. Both MK and KP have made significant contributions to the analysis of research in this review and drafting and revising the review for intellectual content. Both MK and KP have given final approval of the version to be published.
